# Efficient Generation of Germ Line Transmitting Chimeras from C57BL/6N ES Cells by Aggregation with Outbred Host Embryos

**DOI:** 10.1371/journal.pone.0011260

**Published:** 2010-06-22

**Authors:** Marina Gertsenstein, Lauryl M. J. Nutter, Tammy Reid, Monica Pereira, William L. Stanford, Janet Rossant, Andras Nagy

**Affiliations:** 1 Samuel Lunenfeld Research Institute, Mount Sinai Hospital, Toronto, Ontario, Canada; 2 The Toronto Centre for Phenogenomics, Toronto, Ontario, Canada; 3 The Hospital for Sick Children, Toronto, Ontario, Canada; 4 Institute of Biomaterials and Biomedical Engineering, University of Toronto, Toronto, Ontario, Canada; 5 Department of Molecular Genetics, University of Toronto, Toronto, Ontario, Canada; 6 Department of Obstetrics and Gynaecology, University of Toronto, Toronto, Ontario, Canada; University of Birmingham, United Kingdom

## Abstract

Genetically modified mouse strains derived from embryonic stem (ES) cells have become essential tools for functional genomics and biomedical research. Large scale mutagenesis projects are producing libraries of mutant C57BL/6 (B6) ES cells to enable the functional annotation of every gene of the mouse genome. To realize the utility of these resources, efficient and accessible methods of generating mutant mice from these ES cells are necessary. Here, we describe a combination of ICR morula aggregation and a chemically-defined culture medium with widely available and accessible components for the high efficiency generation of germline transmitting chimeras from C57BL/6N ES cells. Together these methods will ease the access of the broader biomedical research community to the publicly available B6 ES cell resources.

## Introduction

The generation of genetically modified mouse strains by homologous recombination in embryonic stem (ES) cells is a major research tool in diverse fields of biology. Historically the majority of manipulations have been performed in ES cells derived from the 129 strain [1] comprised of a wide range of genetically variable substrains not suitable for some studies, in particular immunology [2] and neurobiology [3]. Germline transmission breeding and subsequent backcrosses to C57BL/6 (B6) to generate congenic strains delay functional studies and increase costs, while at the same time genes closely linked to the original targeted locus remain from the original ES cell genome and may confound research results [4]. Together, these considerations led the large scale mouse mutagenesis projects organized under the umbrella of the International Knockout Mouse Consortium (IKMC) to select B6-derived ES cells as the parental lines in which to mutate all protein coding genes of mouse [5], [6]. Improved methods for the generation of germline transmitting chimeric mice from B6 ES cell lines will make the IKMC and other B6 ES cell resources more accessible to the broader biomedical research community.

B6 ES cell lines have been available since the early 1990s [7], [8], [9]; however, in spite of the clear advantages of the inbred background few targeted mutations have been published compared to the widely used 129 ES cell lines [1]. B6 ES cells appear to be less efficient in generating germline transmitting chimeras [10], [11], [12], [13]. A recent report with a large number of clones showed only about 43% *vs.* 81% of germline transmitting clones for B6 *vs.* 129S5 ES cells [14]. B6 ES cells have been shown to be more difficult to maintain in culture [10], [15] and more likely to become aneuploid suggesting instability and deterioration in standard culture conditions [1]. Furthermore microarray analysis of gene expression patterns in cultured 129- and B6-derived ES cells showed that B6 ES cells have a greater tendency to lose their pluripotency in culture in standard 15% FBS LIF-supplemented ES cell medium than 129 ES cells [16].

Various methods have been employed to address the apparent specific requirements of B6 ES cells and to improve methods for their derivation and culture. The use of RESGRO™ medium (Millipore) almost doubled the efficiency of B6 ES cell derivation and generation of germline chimeras and completely ES cell-derived animals by aggregation with tetraploid embryos [17]. The use of optimized KnockOut™ DMEM medium and chemically defined KnockOut™ Serum Replacement (KOSR; Invitrogen) also facilitated the derivation of karyotypically stable and germline competent B6 ES cells [18]. The addition of various signal transduction pathway inducers and inhibitors have resulted in further improvements in ES cell culture and chimera production in various mouse strains including those previously thought to be non-permissive [19], [20], [21], [22]. It is currently postulated that this system of inhibitors constitutes a generic culture condition for the maintenance of authentic pluripotency [23]. Here, we describe a KOSR-based medium for the efficient generation of germline chimeras using our B6-derived ES cell line, designated C2, and its targeted subclones.

A variety of host embryos and methods have been used to generate germline chimeras from B6 ES cells such as BALB/c and C57BL/6-*Tyr^c-2J^*/J (albino B6/J) blastocyst injection [7], [8], [12], [18], [21], [24], [25] and outbred CD-1 [10] and Swiss Webster (SW) [17] morula aggregation. Laser-assisted microinjection into 8-cell embryos has yielded completely ES cell-derived animals [26]. Efficient germline transmission from IKMC targeted B6 ES cell clones was demonstrated recently by Pettitt and colleagues [27] using C57BL/6-*Tyr^c-Brd^* blastocyst injections. However, C57BL/6-*Tyr^c-Brd^* animals are not easy to obtain; similar C57BL/6-*Tyr^c-2J^* mice available from The Jackson Laboratory have a relatively high cost and often require the maintenance of an in-house breeding colony; finally, the classical BALB/c host strain is not very efficient for producing a good number of quality embryos. For these reasons, alternative host embryos for B6 ES cells have been investigated, *e.g.* C3H×BALB/c [28].

Aggregation of cleavage stage embryos with ES cells [29] offers an accessible method for generating ES cell chimeric mice. Since the early 1990s we have exclusively used the aggregation method to generate multiple genetically modified mouse strains from 129S1×129X1 (R1) [30] and 129S6B6F1 (G4) [31] ES cells. Here, we show that ICR morula aggregation is also an efficient method for producing germline transmitting chimeras from B6N ES cell lines. Taken together, the improved culture medium and a more accessible technique for generating chimeras will improve accessibility of the IKMC and other B6 ES cell resources to the broader biomedical research community.

## Results

### Conditioned ES cell medium significantly improves chimera production from B6-derived C2 ES cells

Blastocyst injection is the most common method for producing mouse ES cell chimeras. 8-cell or morula microinjections can be performed with the conventional technique routinely used for blastocysts [32], [33] as well as by introducing a slit cut or hole in the zona pellucida using a glass needle [34], [35], piezo drill [36], [37] or laser [26]. Both blastocyst and 8-cell stage embryos have been used to generate chimeric mice with B6 ES cells [12], [21], [26]. Microinjections require specialized equipment and skilled personnel. To test whether the more accessible method of morula aggregation could efficiently generate B6-derived mouse ES cell chimeras, we attempted morula aggregation using the B6N-derived cell line C2.

C2 ES cells were derived using standard ES cell medium (FBS-DMEM) and methods [38] and were aggregated with ICR embryos to generate chimeras. Although a number of male chimeras with significant coat colour contribution were generated, a poor rate of germ line transmission was observed even at early passage (data not shown) corresponding to the original findings of Auerbach *et. al.* [10] with their B6-derived ES cells. RESGRO™ is an FBS-based medium conditioned by rabbit fibroblast cells transduced with genomic rabbit LIF and shown to be efficient for derivation and generation of germline chimeras using several mouse strains including B6 [17]. Culture of parental C2 ES cells and its transgenic subclones in RESGRO™ resulted in higher numbers of chimeras, increased degree of chimerism and efficient germline transmission (data not shown). Taken together, these observations led us to hypothesize that culture conditions have a major impact on the efficiency of chimera generation with B6 ES cells. To quantitatively assess the effect of culture medium on the production of chimeras from B6-derived ES cells, we undertook a comparison of different culture media. The four culture media tested were standard FBS-DMEM, where the FBS had been lot selected based on the ability to support completely ES cell-derived embryo and pup formation with F1 hybrid-derived (G4) ES cells in the tetraploid complementation assay; RESGRO™ medium (Millipore); VGB6 conditioned medium, a proprietary formulation generously provided by Regeneron Pharmaceuticals, Inc. (Tarrytown, NY); and a defined medium comprised of KnockOut™ DMEM (Invitrogen) with KnockOut™ Serum Replacement (Invitrogen) and supplemented with two inhibitors (2i) [20], [21] (KOSR+2i; see Materials and Methods). The culture media were tested by passaging parental C2 ES cells in the media and then testing their ability to generate chimeric animals based on coat colour contribution.

C2 ES cells were passaged in RESGRO™ medium for several passages (Table 1). After at least three passages, ES cells were aggregated with ICR morula to produce chimeric animals. Consistent with previous reports [1] and our own unpublished data, increasing passage number resulted in a decrease in the chimeric potential of C2 cells with the proportion of >50% chimeric males (as judged by coat colour) decreasing from 32% to 10% among live born animals in four passages (*p* = 0.1274, Fisher's exact test) and the number of >50% chimeric males decreased from 6 to 2 (Table 1).

**Table 1:** Generation of chimeras by ICR morula aggregation from C57BL/6NTac-C2 ES cells cultured in different media.

						No. >50% male
	Medium 1	Medium 2	Medium 3	No. embryos	No. pups born	chimeras (% of
Passage†	(No. passages)	(No. passages)	(No. passages)	transferred	(% of embryos)	live born)
11	RESGRO™ (7)	-	-	100	19 (19%)	6 (32%)^a^
12	RESGRO™ (8)	-	-	200	45 (23%)	7 (16%)
14	RESGRO™ (7)	RESGRO™ (3)	-	176	20 (11%)	2 (10%)^a,c^
14	RESGRO™ (7)	FBS (3)	-	51	20 (39%)	0 (0%)^b,c^
14	RESGRO™ (7)	VGB6 (3)	-	36	6 (17%)	3 (50%)^b^
12	VGB6 (3)	FBS (4)	-	96	32 (33%)	1 (3%)^d,e^
12	VGB6 (3)	RESGRO™ (4)	-	80	20 (25%)	2 (10%)^d,f^
12	VGB6 (3)	VGB6 (4)	-	80	13 (16%)	7 (54%)^e,f^
15	VGB6 (3)	FBS (3)	FBS (3)	165	37 (22%)	0 (0%)^h^
15	VGB6 (3)	FBS (3)	VGB6 (3)	119	25 (21%)	17 (68%)^g,h^
15	VGB6 (3)	VGB6 (3)	VGB6 (3)	67	11 (16%)	5 (45%)^g,h^

† C2 cells derived and cultured in FBS-DMEM until passage 4 when used for pool expansion in RESGRO or passage 5 when used for pool expansion in VGB6.

Fisher's exact test was used for pair-wise comparisons ^a^ *p* = 0.1274; ^b^ *p* = 0.0077; ^c^ *p* = 0.4872; ^d^ *p* = 0.5511; ^e^ *p* = 0.0003; ^f^ *p* = 0.0135; ^g^ *p* = 0.2725. Chi square test was used for 3×2 comparison ^h^ *p*<0.0001.

The expense of RESGRO™ medium limits its use for applications like gene targeting that require large amounts of medium during selection, subcloning, and expansion. To mimic the various culture conditions used in ES cell targeting, we passaged cells sequentially in combinations of RESGRO™, VGB6 medium, or FBS-DMEM. As few as three passages in FBS-DMEM can completely abrogate ES cell contribution to chimeric mice enabled by the previous growth in RESGRO™ medium (Table 1). This can however be rescued by as few as three passages in VGB6 medium (Table 1). Of note, is that no statistically significant difference in the proportion of chimeras born among pups was observed after culture in RESGRO™ or FBS-DMEM before aggregation (*p* = 0.551, Fisher's exact test), while a difference was observed between these two media and culture in VGB6 medium with passage 12 C2 cells (Table 1; *p* = 0.0135 and *p* = 0.0003, respectively, Fisher's exact test). These data enabled us to develop a cost-effective gene targeting approach with the media-intensive selection, subcloning and expansion for screening and cryopreservation done in FBS-DMEM followed by passage of targeted clones in conditioned medium before aggregation.

### Targeted ES cell clones will contribute to the germline of chimeric mice after passage in conditioned medium

C2 ES cells, from a pool expanded in VGB6 conditioned medium generously provided by Regeneron Pharmaceuticals, Inc., were cultured and manipulated in FBS-DMEM during manipulations for gene targeting. After PCR screening to identify targeted subclones, positive clones were each thawed into FBS-DMEM, expanded in 1–2 passages and frozen in vials. After genotype confirmation of expanded clones, cells were thawed in RESGRO™, VGB6 or KOSR medium, passaged three to four times and used for aggregation with ICR embryos. While some clone-to-clone variation was seen with respect to the number and degree of chimerism among progeny (Table S1), on average targeted clones cultured in RESGRO™ resulted in lower numbers and proportion of chimeric mice among live-born pups and a lower number of chimeric males with >50% coat-colour contribution than VGB6-cultured ES cell chimeras (Table 2). Survival of chimeras from birth to weaning did not differ significantly between these two media (*p* = 0.1119, Fisher's exact test). Most important, RESGRO™- and VGB6-cultured ES cells showed identical patterns of germline transmission for these five targeted clones with similar frequencies of germline transmission among tested chimeras (5/18 and 9/17, respectively; *p* = 1.0000, Fisher's exact test). In these experiments we also evaluated KOSR medium for the ability to generate high-contribution and germline transmitting chimeras as it was previously shown to improve the derivation and pluripotency of B6 ES cells [18]. Of the five clones tested, only one transmitted through the germline of chimeric mice after culture in KOSR medium (Table S1). The advantage of chemically-defined media is exemplified by the lot-to-lot variation that can be seen among FBS and conditioned-media lots (unpublished data) which can be compounded by the scarcity of supply and expense in testing and use of these media. Given that KOSR medium enabled germline transmission of one targeted C2 clone and serves as the basal medium for conditioned VGB6 medium [26], we decided to test this as a basal medium supplemented with 2i inhibitors [20] to develop a chemically defined ES cell medium for production of high-level chimeras and good germline transmission rates.

**Table 2:** Generation of chimeras by ICR morula aggregation and germline transmission from 5 targeted C2 subclones cultured in parallel in RESGRO™ or VGB6 medium.

	RESGRO™	VGB6
Average no. embryos transferred	70	62
Average no. pups born (% of embryos)	16.4 (23%)^a^	8.2 (13%)^a^
Average no. chimeras born (% of live born)	4.4 (27%)^b^	5.6 (68%)^b^
Average no. male chimeras weaned (% of chimeras born)	2.8 (64%)^c^	3.4 (61%)^c^
Average no. >50% chimeric males (% of live born)	2.6 (16%)^d^	2.8 (34%)^d^
GLT rate for clones^e^	60%	60%
GLT rate for genes^e^	60%	60%

Fisher's exact test was used for pair-wise comparisons as indicated by superscripts: ^a^ *p* = 0.0009; ^b^ *p*<0.0001; ^c^ *p* = 0.1119; ^d^ *p* = 0.0359.

e Percent germline transmission rate calculated as a proportion of those clones that produced at least one >50% male chimera for testing.

### KOSR+2i enables highly efficient generation of germline transmitting chimeras from targeted B6N-derived C2 ES cells

Several groups have reported that the use of inhibitors of the WNT, FGF and ERK signal transduction pathways significantly improved both the derivation of new ES cell lines, including those from C57BL/6, and their subsequent potential to contribute to chimeric mice and germline transmission [20], [39], [40]. This so-called 3i medium (supplemented with the FGF receptor inhibitor SU5402, the MEK inhibitor PD184352, and the GSK3 inhibitor CHIR99021) is now commercially available (iSTEM Mouse ES cell Media, Stem Cell Sciences, Ltd.). Work with rat ES cells demonstrated that the use of two inhibitors (2i; MEK inhibitor PD0325901 and GSK3 inhibitor CHIR99021) resulted in rat ES cells that were more stable by karyotype analysis and had increased ability to sex-convert host embryos than those derived and cultured with 3i medium [41], [42]. The use of 2i-supplemented medium has also allowed the successful derivation of germline competent NOD mouse ES cells [22]. Our limited success with KOSR reported above and the use of KOSR for both deriving B6 ES cell lines and generating chimeras [18] led us to hypothesize that the addition of 2i to KOSR would improve the ability of ES cells to contribute to chimeric mice and germline transmission potential. To evaluate this, we supplemented KOSR medium with 1 µM of the mitogen-activated protein kinase inhibitor PD0325901 and 3 µM of the glycogen synthase kinase-3 inhibitor CHIR99021 (KOSR+2i) and undertook experiments similar to those described above using 26 targeted subclones of C2 ES cells. We then compared the effects of ES cell culture in KOSR+2i and VGB6 media on the generation of germline transmitting chimeric mice.

As with RESGRO™ and VGB6 media, we observed clone-to-clone variation between KOSR+2i- and VGB6-cultured ES cells (e.g. N00005, N00086, N00343; Table S2). Table 3 shows that on average, aggregation with cells cultured in KOSR+2i resulted in a similar number of pups being born (17.5 *versus* 15.0, *p* = 0.0052, Fisher's exact test), but the number and proportion of chimeras born (scored as black-eyed pups at birth) was higher with cells cultured in VGB6 medium (8.4 and 52% *cf.* 9.7 and 70%, *p*<0.0001, Fisher's exact test). However, the better survival of chimeras born after ICR morula aggregation with KOSR+2i-cultured ES cells compared to VGB6-cultured cells (8.0 and 96% *cf.* 7.2 and 74%, *p*<0.0001, Fisher's exact test) resulted in approximately the same average number of male chimeras being weaned (Table 3, 6.2±3.9 and 6.3±4.6, respectively). On average, more of those chimeras were scored at >50% ES cell contribution by coat colour among live born pups from VGB6-cultured than KOSR+2i-cultured ES cells (5.8 or 37% and 4.3 or 24% of pups born, respectively; *p*<0.0001, Fisher's exact test). The male∶female ratio of KOSR+2i-cultured ES cell chimeras was 3.4∶1 compared to 7.5∶1 for VGB6-cultured ES cell chimeras, suggesting that the VGB6-cultured cells had a higher contribution potential. In spite of this, overall germline transmission rates were similar between the two media (Table 3).

**Table 3:** Generation of chimeras by ICR morula aggregation and germline transmission from 26 targeted C2 subclones cultured in parallel in KOSR+2i or VGB6 medium.

	KOSR+2i	VGB6
Average no. embryos transferred	80	81
Average no. pups born (% of embryos)	17.5 (22%)^a^	15.0 (19%)^a^
Average no. chimeras born (% of live born)	8.4 (48%)^b^	9.7 (65%)^b^
Average no. male chimeras weaned (% of chimeras born)	6.2 (74%)^c^	6.3 (65%)^c^
Average no. >50% chimeric males (% of live born)	4.3 (24%)^d^	5.8 (37%)^d^
No. clones tested	26	26
No. genes tested	23	23
GLT rate for clones^g^	77%^e^	73%^e^
GLT rate for genes^g^	87%^f^	78%^f^

Pair-wise comparisons for statistical analysis are indicated by superscripts: Chi sqare ^a^ *p* = 0.0052; Fisher's exact test ^b,d^ *p*<0.0001; ^c^ *p* = 0.0467; ^e^ *p* = 0.7647; ^f^ *p* = 0.4801.

g Percent germline transmission rate calculated as a proportion of all clones tested, including those that did not produce chimeras.

### Germline transmission is not guaranteed with 100% ES cell derived chimeras

**Figure 1:**
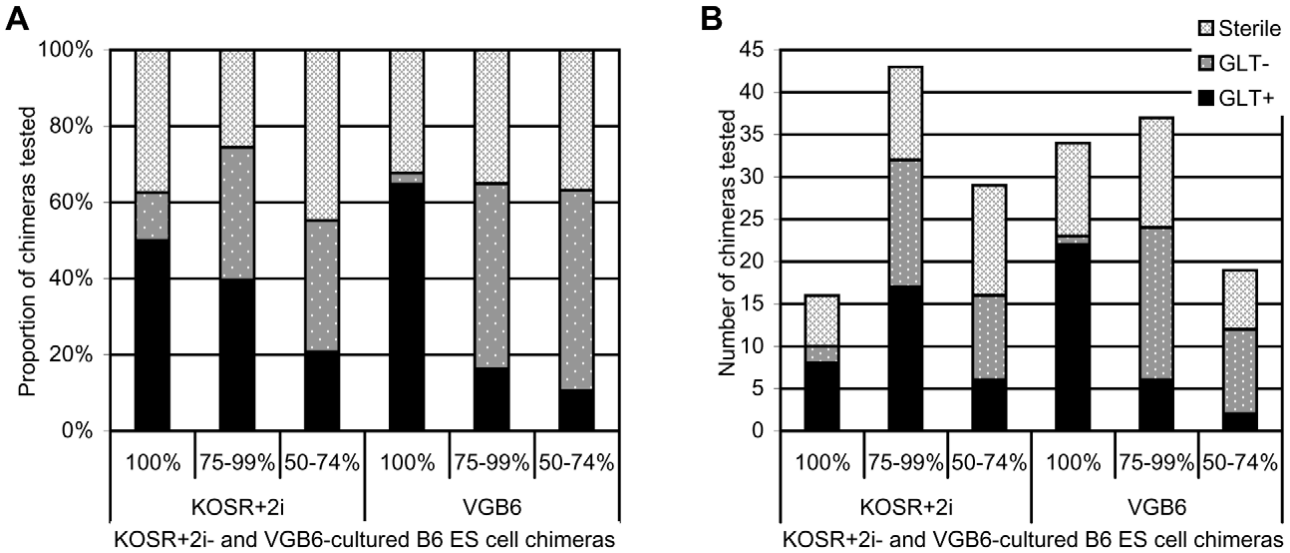
Representation of fertility and germline transmission status of chimeric mice derived from C57BL/6NTac-C2 targeted clones based on degree of coat colour chimerism. **A**. Percentage of mice derived from the same ES cell lines in the same experiments cultured in KOSR+2i or VGB6 prior to aggregation and tested for germline transmission of the ES cell genome. **B**. Number of mice in each category from A.

As shown above, we were able to generate 100% ES cell derived chimeras (as judged by coat colour) using these culture conditions and aggregation method with more 100% by coat colour animals obtained with VGB6-cultured ES cells than with KOSR+2i-cultured ES cells (respectively 2.9 and 1.9 on average per clone, or 19.6% and 11.4% of live born pups, among those clones giving 100% ES cell derived chimeras, *p* = 0.0064, Fisher's exact test; Table S3). For each clone that generated high-level contribution chimeras (>50% by coat colour), we routinely tested up to 6 chimeras for germline transmission of the ES cell genome. As shown in Figure 1, for analysis of germline transmission rates we grouped chimeras according to ES cell contribution as judged by coat colour. Chimeras generated with ES cells cultured in either medium showed a positive correlation between degree of chimerism and germline transmission rates. For both KOSR+2i- and VGB6-cultured ES cells, the decrease in the proportion of tested chimeras showing germline transmission was statistically significant (Figure 1A, *p* = 0.0359 and *p*<0.0001, respectively, Chi square for trend). Among tested chimeras 25–45% were sterile whether ES cells used for aggregation were cultured in VGB6 or KOSR+2i medium (Figure 1A and Table S3; *p*>0.6, Fisher's exact test) and was not correlated with the degree of chimerism (*p* = 0.4328, Chi square for trend). Sterility among ES cell chimeras has been reported and may in some cases be due to the failure of the ES cell to completely sex-convert the chimeric animal, resulting in sterile or hermaphroditic animals [38]. Surprisingly, however, several of the 100% by coat colour ES cell chimeras were fertile but did not transmit the ES cell genome. No difference in either the number or proportion of germline transmitting chimeras was observed for the two media (34% or ^58^/_88_ KOSR+2i and ^59^/_90_ VGB6, *p* = 1.0000, Fisher's exact test). For VGB6-cultured ES cell chimeras germline transmission occurred for 73% (^19^/_26_) of clones tested and 78% (^18^/_23_) of targeted genes; similarly for KOSR+2i-cultured ES cell chimers, germline transmission occurred for 77% (^20^/_26_) of clones tested and 87% (^20^/_23_) of genes (Table 3, Table S2 and Table S3; *p* = 0.7647 and *p* = 0.4801, Fisher's exact test, for clones and genes, respectively).

### KOSR+2i is an appropriate medium for culture of B6-derived ES cell lines

The success of KOSR+2i medium in the culture of several different subclones of targeted C2 ES cells provided strong evidence that this medium would be of wide utility for the culture of B6-derived ES cells and subsequent generation of chimeric mice. At the Toronto Centre for Phenogenomics Transgenic Core, we have implemented the use of KOSR+2i for the generation of chimeric mice using B6-derived ES cells available from the International Knockout Mouse Consortium (IKMC) repositories (see http://www.knockoutmouse.org for more information). These experiments are performed on request as a service to the research community. In addition to the NorCOMM clones used in the experiments above, we have aggregated another 8 targeted clones and 1 gene trap clone (7 genes) from IKMC resources. These clones were cultured in VGB6 and/or KOSR+2i media for at least three passages followed by the production of aggregation chimeras. Germline transmission of 8 of 9 clones was obtained; with 4 of 6 clones cultured in KOSR+2i producing germline-transmitting chimeras (Table 4).

**Table 4:** Generation of germline transmitting chimeras by ICR morula aggregation with targeted B6N ES cell subclones cultured in different types of media.

		No. GLT/No.	clones aggregated^a^
Source of targeted ES cell clones	Parental ES cell line	KOSR+2i	VGB6
KOMP	JM8.N4	2/3	2/3
KOMP	JM8	*nd*	1/1
KOMP	VGB6	1/2	1/3
EuCOMM	JM8.F6	*nd*	1/1
TIGM	B6	1/1	*nd*
NorCOMM	C2	20/26	22/31

*nd*, not done.

a Number of clones aggregated includes those for which no >50% chimeric males were recovered.

## Discussion

The genetic background of mouse strains can affect the phenotype of mutant lines and must therefore be carefully considered when interpreting data from mouse models of disease and development. Using ES cell lines of the same genetic background as the mouse model to be developed simplifies analyses and reduces the time and cost involved in producing appropriate mouse models. Historically, B6 ES cell lines have not been widely used due to reports of genomic instability and a tendency to differentiate in culture [10], [11], [12], [13]. Here we applied the extensive work improving the derivation of new ES cell lines from strains previously identified as non-permissive [20], [21], [22] to develop a defined culture medium, with widely available and accessible components, that enables high efficiency generation of B6 ES cell chimeric mice and subsequent germline transmission. We demonstrate that culture media can significantly affect the ability to generate high-contribution chimeras and that this KOSR+2i medium can be used to improve or rescue cell lines that have been exposed to suboptimal culture conditions (Table 3), restoring their ability to generate high-level chimeras as judged by coat colour. These results are similar to those using 3i medium reported during the preparation of this manuscript [40] and described for C57BL6/J-derived ES cell lines using a GSK3 inhibitor [43]. To date we have generated germline transmitting chimeras for targeted and trapped B6 ES cell clones from six different B6-derived parental lines. These data suggest that this KOSR+2i medium can be of widespread use for the derivation of germline transmitting B6 ES cell chimeras.

Clone to clone variation exists both in the ability to derive high-contribution chimeras and the number and quality of chimeras derived from the same cell line in the different media tested (Table S1 and Table S2). In our limited experience, we have found that repeating the experiment with the same clone to generate more chimeric mice will often enable recovery of germline transmitting chimeric mice. However, our preference is to repeat the experiment with an independent clone for the same targeted or trapped gene, if another is available.

Blastocyst microinjection of albino B6 or BALB/c embryos has been broadly applied for the production of chimeric mice with B6 ES cells. Morula and 8-cell microinjection have also been used over the years to generate chimeras [32], [33], [34], [35]. In fact it was demonstrated that although fewer pups develop to term after morula injection, a higher percentage of them represent high contribution chimeras with higher frequencies of germline transmission compared to injections into blastocyst host embryos [32], [34], [35], [44]. These results demonstrated the role of the developmental stage of host embryos that arguably may be even more important than optimal combinations of ES cell and host embryo strains [25], [45]. Recent developments in morula microinjection techniques include the use of a piezo drill [36], [37] and laser [26] with the last report demonstrating the derivation of completely ES cell derived mice using 8-cell subzonal injection. Convential microinjection of morula also produced completely ES cell derived mice, as judged by coat colour [40]. It is important to note that in these reports of completely ES cell derived mice, particular attention is paid to ES cell culture media [26], [40], [43]. All microinjection protocols require expensive specialized equipment and highly trained operators. Aggregations do not require sophisticated microinjection equipment, only a stereomicroscope and standard embryo manipulation techniques. Inexpensive outbred albino stocks are readily available and provide sufficient numbers and quality of embryos for most purposes. Optimal embryo culture conditions during aggregations are of utmost importance as zona-free embryos are more sensitive and aggregates are cultured longer than injected blastocysts. Aggregation and morula/8-cell microinjection methods are not dependent on the specific developmental stage of the blastocyst suitable for injections. Our, and recently published [43], data show that the aggregation method produces results comparable to those obtained with laser-assisted [26] or conventional subzonal microinjection of 8-cell stage embryos [40] as long as ES cells are maintained in optimal culture conditions. Hence we conclude that aggregation is an efficient and accessible method for the generation of chimeras for establishing new mutant mouse lines from B6 ES cells.

Based on the recent report of microinjection of 3i-cultured B6 ES cells into 8-cell stage ICR embryos to generate germline transmitting chimeras [40], it is possible that conventional or any other method of injection into 8-cell or other E2.5 stage outbred albino embryos will produce similar results as long as attention is paid to the culture conditions of B6 ES cells. B6 ES cells seem to do better in the chemically defined KOSR basal medium rather than FBS-DMEM that exhibits lot-to-lot variation that cannot be controlled when cells are transferred between production centres, repositories and receiving laboratories. Supplementation of KOSR medium with 2i provides an accessible and available alternative to scarce and/or costly proprietary media.

In conclusion, the methods and reagents reported here will make the IKMC and other B6 ES cell resources more accessible to the broader biomedical research community, reducing the costs and time associated with the development of new models for the study of disease and development.

## Materials and Methods

### Ethics Statement

All animal work was carried out following Canadian Council on Animal Care guidelines for Use of Animals in Research and Laboratory Animal Care under protocols approved by the Toronto Centre for Phenogenomics Animal Care Committee; animal use protocol numbers 11-01-0008a-H and 10-11-0153-H.

### Mice

C57BL/6NTac mice were obtained from Taconic for derivation of ES cells. ICR (Harlan) outbred albino stock was used as embryo donors for aggregation with ES cells, CD-1 (Charles River) females were used as pseudopregnant recipients. Animals were maintained on 12 h light/dark cycle and provided with food and water *ad libitum* in individually ventilated units (Techniplast) in the specific-pathogen free facility at the Toronto Centre for Phenogenomics.

### Derivation of ES cells

B6 ES cells were established following the standard methods [38]. Briefly, three week old C57BL/6NTac females were superovulated and mated, and E2.5 embryos were collected from the oviducts and cultured for two days in microdrops of KSOM with amino acids (Millipore) under mineral oil at 37°C in 94% air 6% CO_2_. After 48 h culture, expanded blastocysts were plated individually in 4-well plates (Nunc) containing mitomycin-C (Sigma) treated mouse embryonic fibroblasts (MEF) and FBS-DMEM ES cell medium consisting of high glucose DMEM supplemented with 15% FBS (Hyclone, tested to support generation of chimeras from 129 ES cells), 2 mM glutamine, 1 mM Na Pyruvate, 0.1 mM non-essential amino acids, 50 U/ml penicillin and streptomycin (all from Invitrogen), 0.1 mM 2-mercaptoethanol (Sigma) and 1000 U/ml LIF (prepared with LIF producing plasmid) [46]. Five days after plating, ICM outgrowths were transferred into 30 µl of 0.05% Trypsin-EDTA (Invitrogen), dissociated using a mouth pipette with pulled glass capillary and plated on MEF. Undifferentiated ES cell colonies were then gradually expanded to establish ES cell lines. Lines were selected based on undifferentiated morphology, the presence of Y chromosome by PCR and normal karyotype by chromosome counting [38].

### ES cell culture

ES cells were grown at 37°C in 95% air 5% CO_2_ on MEF obtained from TgN(DR4)1Jae/J mice (http://jaxmice.jax.org/strain/003208.html) at all times except one passage on gelatinized tissue culture plates before aggregation based on standard protocols [38]. Cells were fed daily and passaged when they reached 70–80% confluency. 0.05% Trypsin-EDTA (Invitrogen) was used for the passage of cells grown in FBS-DMEM and Accutase (Millipore SF006) for ES cells grown in KnockOut™ Serum Replacement (SR). Four types of media were used: 1). FBS-DMEM ES cell medium as described above but supplemented with 2 mM GlutaMAX™ (Invitrogen) instead of L-glutamine, 500 U/ml LIF (Millipore ESG1107), 15% FBS (Invitrogen, lot #421412). 2). RESGRO™ (Millipore) medium used for subcloning according to the manufacturer instructions. 3). VGB6 medium containing 15% SR was conditioned by L-cells (ATCC # CRL-2647) and generously provided by Regeneron Pharmaceuticals, Inc. (Tarrytown, NY) [26]. 4). KOSR+2i medium consisted of KnockOut™ DMEM supplemented with 15% KnockOut™ Serum Replacement, 1 mM Na Pyruvate, 0.1 mM non-essential amino acids, 0.1 mM 2-mercaptoethanol, 4 mM GlutaMAX™, 50 U/ml penicillin/streptomycin (all from Invitrogen), 200 U/ml LIF (Millipore ESG1107), 5 µg/ml Insulin (Sigma I0516), 1 µM of the mitogen-activated protein kinase inhibitor PD0325901 (StemGent 04-0006) and 3 µM of the glycogen synthase kinase-3 inhibitor CHIR99021 (StemGent 04-0004).

### Targeting C2 ES cells

Targeting constructs and targeted ES cell lines were generated by the NorCOMM Project and are available at the Canadian Mouse Mutant Repository (www.cmmr.ca). Briefly, approximately 2×10^6^ C2 ES cells were thawed onto a 6-mm tissue culture-treated dish containing irradiated MEFs, expanded over three passages, trypsinized using 0.05% Trypsin (Invitrogen) and centrifuged to remove media. Cells were washed once with electroporation buffer (Millipore), centrifuged and resuspended in electroporation buffer. 8×10^6^ C2 ES cells were transfected with 5 µg of linear DNA in a total volume of 750 µL in a 4-mm electrode gap cuvette using Biorad Gene Pulser (250 V, 500uF Capacity and 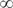 OhMs), chilled on ice for 5 minutes and then plated onto a 100-cm tissue culture-treated dish with 1×10^6^ MEF/plate in FBS-DMEM. Selection with 50–100 µg/ml of G418 was started 24 h post-transfection. After 9–10 days of selection with G418, depending on the construct, colonies were picked and replicated for cryopreservation and DNA analysis.

High throughput genotyping was performed by 96-well PCR using Phire polymerase (NEB) with a gene specific primer 5′ to the end of the 5′ homology arm and an internal vector primer. Once correct homologous recombination of the short arm was confirmed three clones for each vector were expanded in FBS-DMEM. Cells were cryopreserved and DNA was isolated for confirmation of expanded clones prior to aggregation. Long range PCR was performed using Expand Long enzyme (Roche) with a gene specific primer 3′ of the end of the 3′ homology arm and two short internal PCRs were done using Phire polymerase (NEB) to confirm the internal sequences critical to the function of the targeted trap cassette. After sequence confirmation of all four PCR products, one clone was selected for aggregation. Cells were thawed into one of the media formulations described above and passaged three to four times prior to aggregation.

### Generation of chimeras

Details of morula aggregation can be found in [38]. Briefly, embryos were collected at day E2.5 from superovulated ICR (Harlan) female mice. Zonae pellucidae of embryos were removed by treatment with acid Tyrode's solution (Sigma). ES cell colonies were briefly treated with 0.05% Trysin-EDTA or Accutase to form loosely connected clumps of 7–10 cells. Each zona-free embryo was aggregated with a clump of 7–10 cells inside depression wells made in the plastic dish with an aggregation needle (BLS Ltd, Hungary) and cultured overnight in microdrops of KSOM with amino acids (Millipore MR-121D) under mineral oil at 37°C in 94% air/6% CO_2_. The next morning morulae and blastocysts were transferred into the uteri of 2.5-days post-coitum (dpc) pseudopregnant CD-1 (Charles River) females previously mated with vasectomized males. Chimeras were identified at birth by the presence of black eyes and later by coat pigmentation. Chimeric males with more than 50% coat colour contribution were individually bred with ICR females. Tissues from ES cell derived pups identified by eye pigmentation at birth were collected for genotype confirmation and chimera-transmitters were immediately mated with C57BL/6N females to establish mutant strains on B6 background.

### Statistical Analysis

The statistical significance of differences in the proportion of pups or chimeras born after ICR morula aggregation of ES cells cultured under different conditions was calculated using Fisher's exact test for 2×2 tables (pair-wise comparisons) or Chi square test for 2×3 tables (series comparisons) in Prism 5 (GraphPad Software, Inc.). A *p* value≤0.05 was deemed to indicate a statistically significant difference.

## Supporting Information

Table S1 Number, chimerism and GLT data for mice derived from targeted C57BL/6NTac-C2 clones cultured in either RESGROTM, VGB6, or KOSR immediately before aggregation. Raw data for Table 2.
(0.01 MB PDF)

Table S2 Number, chimerism and GLT data for mice derived from targeted C57BL/6NTac-C2 clones cultured in either KOSR+2i or VGB6 immediately before aggregation. Raw data for Table 3.
(0.02 MB PDF)

Table S3 Germline transmission test results for individual chimeric mice derived from targeted C57BL/6NTac-C2 clones. Raw data for Figure 1.
(0.01 MB PDF)
