# Supplementary material for: Efficient Generation of Germ Line Transmitting Chimeras from C57BL/6N ES Cells by Aggregation with Outbred Host Embryos
Source: PLoS One. 2010 Jun 22;5(6):e11260. doi: 10.1371/journal.pone.0011260 (PMC2889837; doi:10.1371/journal.pone.0011260)
Supplement: Table S1 — Number, chimerism and GLT data for mice derived from targeted C57BL/6NTac-C2 clones cultured in either RESGROTM, VGB6, or KOSR immediately before aggregation. Raw data for Table 2. (0.01 MB PDF) [file pone.0011260.s001.pdf]

Table S1. Number, chimerism and GLT data for mice derived from targeted C57BL/6NTac-C2 clones cultured in either RESGRO^{TM}, VGB6, or KOSR immediately before aggregation.

				Total #	Development					Weaned										
				aggregates	Born		Chimeras			M		F	%chim	Males' ES coat colour contribution				Fertility		
Construct	Gene Name	Clone ID	Media	transferred	#	%	#	%born	%total	#	%	#	>50%	100%	99-75%	74-50%	<50%	# BP	# GLT	%
N00006	Mcm10	B03	RESGRO	57	13	22.8%	7	53.8%	12.3%	6	86%	1	71%	2	1	2	1	6	2	33%
N00006	Mcm10	B03	VGB6	59	4	6.8%	3	75.0%	5.1%	2	67%	0	67%	2	0	0	0	2	2	100%
N00006	Mcm10	B03	KOSR	41	14	34.1%	8	57.1%	19.5%	7	88%	0	63%	0	4	1	2	5	2	40%
N00007	Anxa6	F05	RESGRO	57	20	35.1%	7	35.0%	12.3%	4	57%	0	43%	1	1	1	1	7	2	29%
N00007	Anxa6	F05	VGB6	60	12	20.0%	6	50.0%	10.0%	4	67%	0	50%	0	3	0	1	3	2	67%
N00007	Anxa6	F05	KOSR	57	11	19.3%	2	18.2%	3.5%	1	50%	1	0%	0	0	0	1	1	0	0%
N00009	Ahcy	F11	RESGRO	82	2	2.4%	2	100.0%	2.4%	0	0%	2	100%	0	0	2	0	2	0	0%
N00009	Ahcy	F11	VGB6	63	7	11.1%	5	71.4%	7.9%	1	20%	2	0%	0	0	0	1	1	0	0%
N00009	Ahcy	F11	KOSR	81	33	40.7%	4	12.1%	4.9%	3	75%	1	0%	0	0	0	3	3	0	0%
N00016	Centd2	A05	RESGRO	86	21	24.4%	5	23.8%	5.8%	3	60%	1	40%	0	1	1	1	2	1	50%
N00016	Centd2	A05	VGB6	86	11	12.8%	8	72.7%	9.3%	6	75%	0	75%	4	2	0	0	6	5	83%
N00016	Centd2	A05	KOSR	87	27	31.0%	5	18.5%	5.7%	3	60%	0	60%	0	0	3	0	3	0	0%
N00020	Tbc1d2	B10	RESGRO	69	26	37.7%	1	3.8%	1.4%	1	100%	0	100%	0	0	1	0	1	0	0%
N00020	Tbc1d2	B10	VGB6	42	7	16.7%	6	85.7%	14.3%	4	67%	0	50%	0	3	0	1	4	0	0%
N00020	Tbc1d2	B10	KOSR	84	18	21.4%	1	5.6%	1.2%	0	0%	1	0%	0	0	0	0	NT	NT	NT
