# Supplementary material for: Efficient Generation of Germ Line Transmitting Chimeras from C57BL/6N ES Cells by Aggregation with Outbred Host Embryos
Source: PLoS One. 2010 Jun 22;5(6):e11260. doi: 10.1371/journal.pone.0011260 (PMC2889837; doi:10.1371/journal.pone.0011260)
Supplement: Table S2 — Number, chimerism and GLT data for mice derived from targeted C57BL/6NTac-C2 clones cultured in either KOSR+2i or VGB6 immediately before aggregation. Raw data for Table 3. (0.02 MB PDF) [file pone.0011260.s002.pdf]

Table S2. Number, chimerism and GLT data for mice derived from targeted C57BL/6NTac-C2 clones cultured in either KOSR+2i or VGB6 immediately before aggregation.

				Total #	Development					Weaned										
				aggregates	Born		Chimeras			M		F	%chim	ES coat colour contribution				Fertility		
Construct	Gene	Clone	Media	transferred	#	%	#	%born	%total	#	% born	#	>50%	100%	99-75%	74-50%	<50%	# BP	# GLT	%
N00005	Zfp202	G01	2i	167	24	14.4%	13	54.2%	7.8%	6	46%	4	38%	1	0	4	1	4	3	75%
N00005	Zfp202	G01	VGB6	166	15	9.0%	11	73.3%	6.6%	7	64%	2	45%	1	2	2	2	3	0	0%
N00020	Tbc1d2	A11	2i	72	23	31.9%	10	43.5%	13.9%	9	90%	5	80%	2	2	4	6	4	1	25%
N00020	Tbc1d2	A11	VGB6	70	17	24.3%	12	70.6%	17.1%	9	75%	0	75%	4	5	0	0	4	2	50%
N00020	Tbc1d2	C11	2i	92	20	21.7%	1	5.0%	1.1%	1	100%	2	100%	0	0	1	2	1	0	0%
N00020	Tbc1d2	C11	VGB6	95	15	15.8%	10	66.7%	10.5%	8	80%	1	80%	4	2	2	1	4	2	50%
N01150¶	Zfp871	D01	2i	83	22	26.5%	18	81.8%	21.7%	7	39%	0	22%	1	1	2	3	4	2	50%
N01150¶	Zfp871	D01	VGB6	63	6	9.5%	6	100.0%	9.5%	0	0%	0	0%	0	0	0	0	-	-	-
N01174	Angel1	F12	2i	63	6	9.5%	3	50.0%	4.8%	11	367%	1	33%	0	0	1	2	1	0	0%
N01174	Angel1	F12	VGB6	102	22	21.6%	14	63.6%	13.7%	11	79%	1	57%	4	2	2	0	8	1	13%
N01174	Angel1	D11	2i	77	19	24.7%	7	36.8%	9.1%	7	100%	0	100%	1	4	2	2	5	1	20%
N01174	Angel1	D11	VGB6	79	23	29.1%	9	39.1%	11.4%	8	89%	1	56%	1	2	2	3	6	1	17%
N00086	Dhx40	C07	2i	67	30	44.8%	7	23.3%	10.4%	7	100%	0	43%	0	1	2	4	3	1	33%
N00086	Dhx40	C07	VGB6	51	11	21.6%	9	81.8%	17.6%	6	67%	0	56%	1	2	2	2	3	0	0%
N00105	Itch	A08	2i	68	24	35.3%	4	16.7%	5.9%	5	125%	0	25%	0	0	1	4	1	1	100%
N00105	Itch	A08	VGB6	54	17	31.5%	8	47.1%	14.8%	7	88%	0	63%	1	1	3	2	5	1	20%
N00343	15M20Rik	F05	2i	45	12	26.7%	4	33.3%	8.9%	3	75%	1	25%	0	0	1	3	1	0	0%
N00343	15M20Rik	F05	VGB6	128	19	14.8%	13	68.4%	10.2%	5	38%	4	46%	0	4	2	3	4	1	25%
N00135	Otud7b	G09	2i	47	15	31.9%	8	53.3%	-	5	63%	3	50%	0	2	2	4	4	1	25%
N00135	Otud7b	G09	VGB6	46	12	26.1%	8	66.7%	17.4%	6	75%	1	75%	1	0	5	0	6	2	33%
N01113	Psmd2	F10	2i	24	1	4.2%	1	100.0%	4.2%	1	100%	0	100%	0	0	1	0	1	1	100%
N01113	Psmd2	F10	VGB6	24	2	8.3%	2	100.0%	8.3%	2	100%	0	100%	2	0	0	0	2	2	100%
N01113	Psmd2	F10	2i	69	13	18.8%	8	61.5%	11.6%	7	88%	1	50%	2	2	0	3	4	2	50%
N01113	Psmd2	F10	VGB6	69	17	24.6%	15	88.2%	21.7%	11	73%	4	40%	2	2	2	5	4	2	50%
N01554†	18L17Rik	A03	2i	69	3	4.3%	1	33.3%	1.4%	0	0%	0	0%	-	-	-	-	-	-	-
N01554†	18L17Rik	A03	VGB6	41	3	7.3%	3	100.0%	-	0	0%	0	0%	-	-	-	-	-	-	-
N00324	Senp3	D04	2i	84	17	20.2%	8	47.1%	9.5%	7	88%	5	63%	0	3	2	2	5	3	60%
N00324	Senp3	D04	VGB6	54	13	24.1%	10	76.9%	18.5%	9	90%	0	90%	0	3	6	0	4	1	25%
N00109	Pum1	G01	2i	84	10	11.9%	9	90.0%	10.7%	8	89%	1	67%	0	3	3	3	6	1	17%
N00109	Pum1	G01	VGB6	84	14	16.7%	14	100.0%	16.7%	7	50%	2	43%	2	0	4	3	6	3	50%
N01554	18L17Rik	E03	2i	57	21	36.8%	13	61.9%	22.8%	12	92%	1	92%	5	2	5	0	4	2	50%
N01554	18L17Rik	E03	VGB6	53	12	22.6%	9	75.0%	17.0%	9	100%	0	100%	5	0	4	0	3	3	100%
N01551	Ostf1	A02	2i	73	25	34.2%	20	80.0%	27.4%	19	95%	1	45%	1	8	0	10	3	3	100%
N01551	Ostf1	A02	VGB6	75	12	16.0%	10	83.3%	13.3%	10	100%	0	100%	7	3	0	0	4	2	50%
N01605	Zbtb45	C04	2i	94	22	23.4%	11	50.0%	11.7%	8	73%	3	64%	4	2	1	4	3	1	33%
N01605	Zbtb45	C04	VGB6	88	8	9.1%	7	87.5%	8.0%	5	71%	0	71%	5	0	0	0	3	2	67%

Table S2. Number, chimerism and GLT data for mice derived from targeted C57BL/6NTac-C2 clones cultured in either KOSR+2i or VGB6 immediately before aggregation.

				Total #	Development					Weaned				ES coat colour contribution				Fertility		
				aggregates	Born		Chimeras			M		F	%chim							
Construct	Gene	Clone	Media	transferred	#	%	#	%born	%total	#	% born	#	>50%	100%	99-75%	74-50%	<50%	# BP	# GLT	%
N01234	Zfp428	G10	2i	91	17	18.7%	8	47.1%	8.8%	5	63%	3	38%	1	0	2	5	3	0	0%
N01234	Zfp428	G10	VGB6	ND	-	-	-	-	-	-	-	-	-	-	-	-	-	-	-	-
N00087	Rab1	E05	2i	127	44	26.4%	12	27.3%	9.4%	5	42%	2	50%	0	1	5	5	7	1	14%
N00087	Rab1	E05	VGB6	132	25	13.8%	9	36.0%	6.8%	1	11%	2	22%	0	0	2	4	2	0	0%
N00138	Zbtb8a	A10	2i	105	18	17.1%	13	72.2%	12.4%	7	54%	3	38%	0	1	4	5	5	1	20%
N00138	Zbtb8a	A10	VGB6	75	3	4.0%	3	100.0%	4.0%	1	33%	0	33%	1	0	0	0	1	1	100%
N00279	Pde4d	D08	2i	51	12	23.5%	7	58.3%	13.7%	4	57%	0	57%	3	0	1	0	4	2	50%
N00279	Pde4d	D08	VGB6	97	12	12.4%	7	58.3%	7.2%	6	86%	0	71%	3	1	1	1	4	1	25%
N00352	Htra1	D01	2i	120	14	11.7%	8	57.1%	6.7%	5	63%	1	38%	0	2	1	2	3	1	33%
N00352	Htra1	D01	VGB6	73	6	8.2%	2	33.3%	2.7%	1	50%	1	50%	1	0	0	0	1	1	100%
N00082	Taok1	A02	2i	107	29	27.1%	19	65.5%	17.8%	8	42%	9	32%	0	0	6	2	5	0	0%
N00082	Taok1	A02	VGB6	100	21	21.0%	12	57.1%	12.0%	5	42%	2	42%	0	0	5	0	4	0	0%
N01077	Myd88	C06	2i	127	14	11.0%	2	14.3%	1.6%	1	50%	2	50%	0	0	1	2	1	1	100%
N01077	Myd88	C06	VGB6	133	21	15.8%	4	19.0%	3.0%	1	25%	0	0%	0	0	0	3	-	-	-
N01539	Senp6	C07	2i	63	7	11.1%	6	85.7%	9.5%	5	83%	1	33%	1	0	1	4	2	1	50%
N01539	Senp6	C07	VGB6	82	32	39.0%	19	59.4%	23.2%	8	42%	1	53%	5	1	4	1	4	1	25%
N01590	Tmem116	D08	2i	29	11	37.9%	6	54.5%	20.7%	5	83%	0	67%	1	1	2	1	4	1	25%
N01590	Tmem116	D08	VGB6	73	31	42.5%	25	80.6%	34.2%	21	84%	0	64%	5	0	11	5	5	1	20%

¶Half of KOSR+2i (9) and all VGB6 chimeras (6) died before weaning.

†All chimeras dead PN 3 days.

IP, testing in progress
