# Supplementary material for: Efficient Generation of Germ Line Transmitting Chimeras from C57BL/6N ES Cells by Aggregation with Outbred Host Embryos
Source: PLoS One. 2010 Jun 22;5(6):e11260. doi: 10.1371/journal.pone.0011260 (PMC2889837; doi:10.1371/journal.pone.0011260)
Supplement: Table S3 — Germline transmission test results for individual chimeric mice derived from targeted C57BL/6NTac-C2 clones. Raw data for Figure 1. (0.01 MB PDF) [file pone.0011260.s003.pdf]

Table S3. Germline transmission test results for individual chimeric mice derived from targeted C57BL/6NTac-C2 clones.

TOTAL # CHIMERAS TESTED PER CATEGORY:

Media	100% chimeras	99-75% chimeras	74-50% chimeras
RESGRO	3	6	2
VGB6	6	8	0
2i	16	43	29
VGB6	34	37	19

# CHIMERAS PER GLT CATEGORY:

	100% chimeras			99-75% chimeras			74-50% chimeras		
Media	GLT+	GLT-	sterile	GLT+	GLT-	sterile	GLT+	GLT-	sterile
RESGRO	3	0	0	2	3	1	0	1	1
VGB6	6	0	0	3	2	3	0	0	0
KOSR+2i	8	2	6	17	15	11	6	10	13
VGB6	22	1	11	6	18	13	2	10	7

PERCENTAGES:

	100% chimeras			99-75% chimeras			74-50% chimeras		
Media	GLT+	GLT-	sterile	GLT+	GLT-	sterile	GLT+	GLT-	sterile
RESGRO	100%	0%	0%	33%	50%	17%	0%	50%	50%
VGB6	100%	0%	0%	38%	25%	38%	0%	0%	0%
KOSR+2i	50%	13%	38%	40%	35%	26%	21%	34%	45%
VGB6	65%	3%	32%	16%	49%	35%	11%	53%	37%

GRAND TOTALS:

GLT+	75
GLT-	62
Sterile	66

Total # chimeras tested: **203**
